# Laryngeal Sarcoidosis and Swallowing: What Do We Know About Dysphagia Assessment and Management in this Population?

**DOI:** 10.1007/s00455-021-10305-4

**Published:** 2021-05-26

**Authors:** Lindsay Lovell, Gemma M. Clunie, Chadwan Al-Yaghchi, Justin Roe, Guri Sandhu

**Affiliations:** 1 Department of Otolaryngology, Head and Neck Surgery, National Centre for Airway Reconstruction, Imperial College Healthcare NHS Trust, London, UK; 2 Department of Surgery and Cancer, Imperial College London, London, UK; 3 Speech and Language Therapy Department – Airways / ENT, Charing Cross Hospital, Imperial College Healthcare NHS Trust, Fulham Palace Road, London, W6 8RF UK

**Keywords:** Laryngeal sarcoidosis, Dysphagia

## Abstract

**Introduction:**

Sarcoidosis is a chronic granulomatous disease of unknown aetiology and laryngeal involvement is seen in a small percentage of cases. Dysphagia is a common but under-reported symptom. Little is known about how dysphagia typically presents or is managed in the context of this fluctuating disease. We present our case series using an SLT-led model of assessment and management.

**Methods:**

A literature search was conducted for any articles that reported both laryngeal sarcoidosis and dysphagia. We then analysed a case series of laryngeal sarcoidosis patients treated at Charing Cross Hospital. We report on multidimensional swallowing evaluation and rehabilitative interventions.

**Results:**

Seventeen papers report both laryngeal sarcoidosis and dysphagia, with only one paper giving details on the nature of the dysphagia and the treatment provided.

In our case series (n = 7), patients presented with FOIS Scores ranging from 5 to 7 pre-operatively (median = 6). Aspiration (median PAS Score = 6 and Range = 3–8) and pharyngeal residue were common. Sensory issues were also prevalent with most unaware of the extent of their difficulties. Management interventions included safe swallowing advice, compensatory strategies, exercises and close surveillance given their potential for repeated surgical interventions.

**Conclusion:**

Laryngeal sarcoidosis is a rare condition. Dysphagia is under-reported and our experience highlights the need for specialist dysphagia intervention. Further research is required to understand dysphagia management requirements in the context of this fluctuating disease process.

## Introduction

Sarcoidosis is a chronic granulomatous disease of unknown aetiology. It typically affects patients between 20 and 40 years of age and most commonly affects the lungs, lymph nodes, liver, eyes, skin, bones and nervous system [1]. The disease is characterised by tissue infiltration by mononuclear phagocytes and lymphocytes with associated non-caseating granuloma formation [2]. Sarcoidosis is a fluctuating disease, so patients may have flare-ups and relapses following seemingly successful initial treatment. An auto-immune aetiology has been suggested but research is on-going [1].

Laryngeal involvement is rare. Some authors suggest between 3 and 5% of cases involve the larynx, and it is usually localised to the supraglottic region [3]. It can feature as part of a systemic sarcoid disease process, or present as an isolated lesion in the larynx. Typical treatment may include intralesional and systemic steroids, low-dose radiation, immuno-suppressants and surgical excision. If severe airway obstruction occurs, tracheostomy may be required [1].

In our clinical experience, dysphagia is a common but under-reported symptom. Patients either present with no reported dysphagia symptoms, or will report the feeling of harder foods sticking in the throat, taking longer to eat at mealtimes, and/or coughing on oral intake. Little is mentioned in the literature about how dysphagia typically presents or is managed in the context of this fluctuating disease. The National Centre for Airway Reconstruction at Imperial College Healthcare has one of the largest laryngeal sarcoidosis caseloads in the UK. In this paper, we review the available literature on dysphagia in this population, map it to current practice in our institution, and discuss the need for specialist dysphagia intervention with this patient population. Our hypothesis before carrying out this retrospective data analysis was that patients would have more severe dysphagic symptoms on instrumental swallowing studies than they were self-reporting. The authors can confirm that this is an original study.

## Method

A literature search was performed by the lead author using Medline and EMBASE, for papers that mentioned both laryngeal sarcoidosis and dysphagia, as well as common variations on this terminology (see Table 1). No limits were set on date of publication or language. Papers were excluded if a diagnosis of laryngeal sarcoidosis was not specifically stated. There were no other exclusion criteria.

**Table 1:** Search terms used in literature search

	Database	Search term	Results
1	Medline	(laryng* ADJ3 sarcoid*).ti,ab	72
3	Medline	(swallow*).ti,ab	27,376
4	Medline	(eating).ti,ab	67,184
5	Medline	(drinking).ti,ab	102,587
6	Medline	(deglutition*).ti,ab	2361
7	Medline	("oropharyngeal dysphagia").ti,ab	750
8	Medline	("dysphagia").ti,ab	25,030
9	Medline	"DEGLUTITION DISORDERS"/	19,034
10	Medline	DEGLUTITION/	9198
11	Medline	(3 OR 4 OR 5 OR 6 OR 7 OR 8)	212,908
12	Medline	(9 OR 10)	25,525
13	Medline	(11 OR 12)	220,783
15	Medline	(1 AND 13)	13
16	EMBASE	(laryng* ADJ3 sarcoid*).ti,ab	80
17	EMBASE	(swallow*).ti,ab	41,851
18	EMBASE	(eating).ti,ab	89,935
19	EMBASE	(drinking).ti,ab	134,823
20	EMBASE	(deglutition*).ti,ab	2670
21	EMBASE	("oropharyngeal dysphagia").ti,ab	1381
22	EMBASE	("dysphagia").ti,ab	41,928
23	EMBASE	"SWALLOWING DISORDER"/	62,257
24	EMBASE	(17 OR 18 OR 19 OR 20 OR 21 OR 22)	291,943
25	EMBASE	(23 OR 24)	314,971
26	EMBASE	(16 AND 25)	13

A retrospective analysis was then undertaken of laryngeal sarcoidosis patients who had been referred to a Speech and Language Therapist (SLT) for dysphagia management at Imperial from 2016 to 2019. Any patient who was seen in an Airways Ear, Nose, and Throat (ENT) clinic and mentioned swallowing difficulties was referred to the Airways SLT team for assessment and management. All patients were evaluated with an instrumental swallow assessment – either a videofluoroscopy (VFS) or a Fibreoptic Endoscopic Evaluation of Swallowing (FEES), depending on their clinical presentation. VFS studies were carried out by 2 VFS trained SLTs and were reviewed frame-by-frame, both together and separately, before writing a report and coming to a consensus on scoring. There was no need to consult a third evaluator at any time. Studies were carried out at 30 frames per second [4], following a standardised protocol. Barium sulphate was the usual material mixed with food and drink, unless there was significant concern about aspiration risk, in which case a water-soluble contrast was used instead. Food and fluids were generally administered in order from thin fluids (IDDSI level 0) [5], to puree (IDDSI level 4), to easy chew (IDDSI level 7) and then regular diet (IDDSI level 7), unless information during the assessment suggested a different order would be beneficial. All patients were able to feed themselves without the help of an SLT. For the first fluid bolus, patients were instructed to hold the bolus in their mouth before being given the instruction to swallow. For subsequent fluid boluses, patients were instructed to take a sip or carry out continuous drinking as they normally would, without the need for an oral hold. Puree diet was given on a teaspoon, and when solid food was given, patients were instructed to take a bite that was ‘normal for them’. Images were taken in both lateral and anteroposterior planes. The FEES studies were again carried out by 2 FEES trained SLTs, one acting as the endoscopist and the other as the assessor. Real food was given in the same order of consistencies as for the VFS studies, and scores were agreed on by consensus, after reviewing the study images together. Again, all patients were able to feed themselves and they were given the same instructions as those given in the VFS studies.

**Fig. 1:**
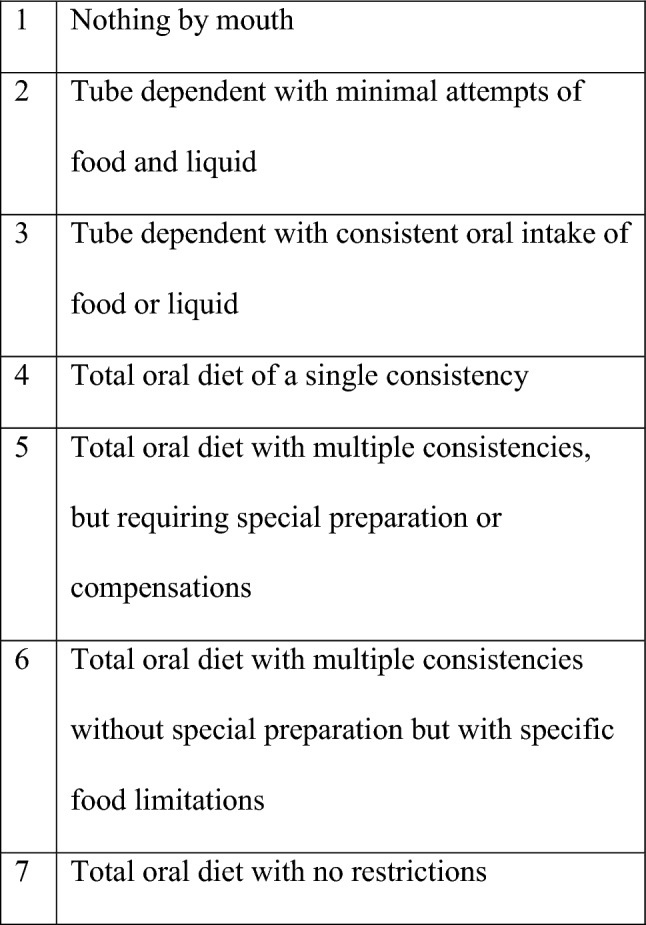
Function Oral Intake Scale (FOIS)

**Fig. 2:**
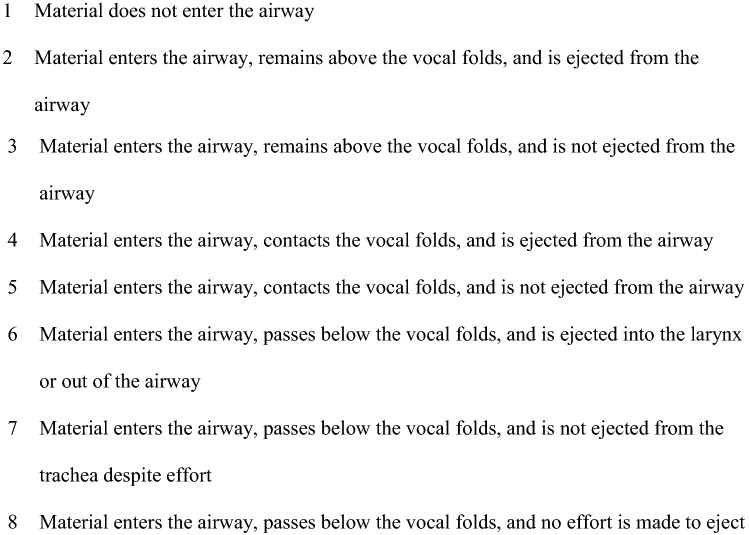
Penetration-Aspiration Scale (PAS)

**Fig. 3:**
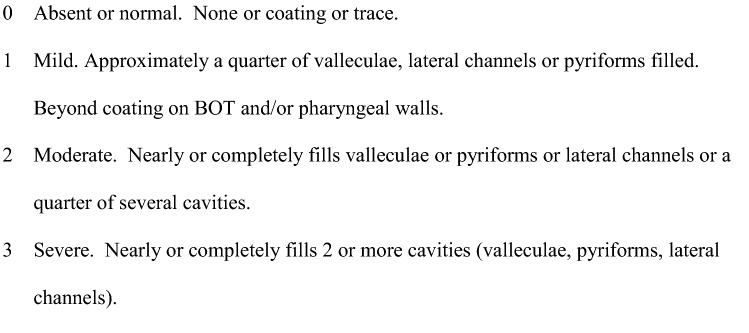
Langmore’s Residue Score

We developed a multidimensional dashboard of swallowing measures including the Functional Oral Intake Scale (FOIS) [6] (see Fig. 1), Penetration-Aspiration Scale (PAS) [7] (see Fig. 2) and Langmore’s Residue Score (see Fig. 3), as part of our standard of care. Sensory issues were assessed by using the PAS score, which describes whether patients were sensate to any penetration or aspiration. Data were extracted from patients’ electronic case notes. This retrospective analysis was approved locally under the Imperial College Healthcare NHS Trust’s ENT Directorate as a service evaluation.

## Results

Seventeen papers which mentioned both laryngeal sarcoidosis and dysphagia were found during the literature search [see Table 2]. Eleven of the papers (65%) mentioned dysphagia as being a symptom of laryngeal sarcoidosis, with no further details given. Five papers (29%) alluded to dysphagic symptoms which resolved following medical treatment. Only one paper gave any details about the dysphagia, and how it was assessed and treated [8]. The authors reported on a case of a 43-year-old woman with progressive dysphagia, who was found to have a cricopharyngeal bar and oesophageal issues on a barium swallow study. An endoscopic cricopharyngeal myotomy was performed, which they report ‘moderately improved her dysphagia’. There were no further data or discussion of the dysphagia in this or any other paper, and no mention of SLT as part of patient management.

**Table 2:** Literature review table of reference

	Paper	Type of study	Dysphagia assessment and management
1	Swain SK, Samal R, Sahu MC. An isolated laryngeal sarcoidosis in a child threatening to the airway – A case report. Pediatria Polska. 2016;91(1):69–72 [9]	Case study	None reported
2	Ketharanathan N, Den Herder C, Veenstra J, De Vries N. Geisoleerde laryngeale sarcoidoselsolated laryngeal sarcoidosis: A case report. Nederlands Tijdschrift voor Keel-Neus-Oorheelkunde. 2006;12(1):23–25 [10]	Case study	None reported
3	Fortune S, Courey MS. Isolated laryngeal sarcoidosis. Otolaryngol Head Neck Surg. 1998;118(6):868–870 [11]	Case study	3-month history of dysphagia to solids. Nil reported assessment or SLT management. Dysphagia improved after medical treatment
4	Sataloff RT, Spiegel JR, Heuer RJ. Laryngeal sarcoidosis and candidiasis. Ear, Nose and Throat Journal. 1995;74(2):77 [12]		Paper unavailable
5	Jakse R, Fleischmann G. Diagnosis and treatment of laryngeal sarcoidosis. HNO. 1985;33(3):118–123 [13]	Case report	None reported
6	Dean CM, Sataloff RT, Hawkshaw MJ, Pribikin E. Laryngeal sarcoidosis. Journal of voice. 2002;16(2):283–288 [1]	Case study	None reported
7	Mayerhoff RM, Pitman MJ. Atypical and disparate presentations of laryngeal sarcoidosis. The annals of otology, rhinology and laryngology. 2010;119(10):667–671 [8]	Case series	4 patients. 1 with progressive dysphagia – barium swallow study showed oesophageal dysmotility and a cricopharyngeal bar. An endoscopic cricopharyngeal myotomy was performed, with moderate improvements to the dysphagia. Nil SLT involvement reported
8	Tsubouchi K, Hamada N, Ijichi K, Umezaki T, Takayama K, Nakanishi Y. Spontaneous improvement of laryngeal sarcoidosis resistant to systemic corticosteroid administration. Respirology case reports. 2015;3(3):112–114 [14]	Case study	Dysphagia resolved after medical treatment
9	Benjamin B, Dalton C, Croxson G. Laryngoscopic diagnosis of laryngeal sarcoid. The annals of otology, rhinology and laryngology. 1995;104(7):529–531 [15]	Case series	None reported
10	Ridder GJ, Strohhaecker H, Loehle E, Golz A, Fradis M. Laryngeal sarcoidosis: treatment with the antileprosy drug clofazimine. The annals of otology, rhinology and laryngology.2000;109(12):1146–1149 [16]	Case study	Dysphagia resolved after medical treatment
11	Krespi YP, Mitrani M, Husain S, Meltzer CJ. Treatment of laryngeal sarcoidosis with intralesional steroid injection. The annals of otology, rhinology and laryngology. 1987;96(6):713–715 [17]	Case series	None reported
12	McHugh K, deSilva M, Kilham HA. Epiglottic enlargement secondary to laryngeal sarcoidosis. Pediatric radiology. 1993;23(1):71 [18]	Case study	6-week history of dysphagia. Nil SLT assessment or management reported
13	Duchemann B, Lavole A, Naccache J-M, Nunes, H, Benzakin S, Lefevre M et al. Laryngeal sarcoidosis: a case–control study. Sarcoidosis Vasc Diffuse Lung Dis. 2014;31(3):227–234 [19]	Retrospective case–control study	None reported
14	Lede Barreiro A, Diaz Arguello JJ, Fernandez Martinez JA, Martinez Ferreras A. Laryngeal sarcoidosis: unique location or first manifestation? Acta otorrinolaringologica espanola. 2012;63(3):230–232 [20]	Case study	None reported
15	Bower JS, Belen JE, Weg JG, Dantzker DR. Manifestations and treatment of laryngeal sarcoidosis. The American review of respiratory disease. 1980;122(2):325–332 [21]	Unknown	None reported
16	Agrawal Y, Godin DA, Belafsky PC. Cytotoxic agents in the treatment of laryngeal sarcoidosis: a case report and review of the literature. Journal of voice. 2006;20(3):481–484 [3]	Case study	None reported
17	Delides A, Sakagiannis G, Maragoudakis P, Gouloumi A-R, Katsimbri P, Giotakis I et al. Dysphagia caused by chronic laryngeal oedema. Dysphagia. 2015;30:583–585 [2]	Case study	Dysphagia resolved after medical treatment

Our retrospective analysis of cases at the National Centre for Airway Reconstruction, Imperial, identified seven laryngeal sarcoidosis patients (out of seventeen being actively managed by ENT), who were suspected of having dysphagia and were managed by a SLT between 2016 and 2019 (41%). Our case series was made up of five systemic and two isolated laryngeal sarcoid cases, five females and two males, with an age range of 21–69 (see Table 3). All seven patients were treated with surgical intervention, often on multiple occasions, which included laser pepperpotting [22], interarytenoid scar division, intralesional steroid injections, trans-glottic balloon dilatation and vocal fold injections for thinned cords.

**Table 3:** Case series patient demographics and swallowing dysfunction

Case no	Gender	Age	Type of sarcoid	Endoscopic surgical interventions	SLT intervention	FOIS pre-op	FOIS post-op	Pre-op VFS	Pre-op FEES	Post-op VFS	Post-op FEES
1	F	56	Systemic—nasal and laryngeal	× 2	Food textures, voice and reflux advice	7	6	Incomplete epiglottic deflection, reduced laryngeal elevation, penetration and aspiration during the swallow. PAS 6	No	No	Scarred and stiff epiglottis, reduced adduction of vocal cords, reduced tongue base retraction and epiglottic deflection, spontaneous clearance of penetration. PAS 2, RS 1
2	M	45	Systemic—nasal, laryngeal and suspected neuro	× 2	Assessment only		7	No	No	Variable swallow trigger. PAS 1	No
3	F	35	Laryngeal	× 3	Safe eating strategies	5		No	Reduced tongue base retraction and pharyngeal squeeze, penetration during and post-swallow. PAS 3, RS 2	No	No
4	F	34	Systemic—pulmonary and laryngeal	× 3	Safe eating strategies, chronic cough and hypersensitivity therapy including reflux management	5		No	Very bulky arytenoids with minimal pyriform space. Hypersensitivity. Penetration and aspiration during and post-swallow. PAS 8, RS 0	No	No
5	F	54	Systemic—pulmonary, nasal and laryngeal	× 1	No swallow therapy as patient wished to focus on voice therapy only	6	5	No	No	No	Delayed swallow trigger, reduced pharyngeal squeeze, penetration during swallow PAS 5, RS 1
6	M	22	Laryngeal	× 1	Pre-op advice. Post-op swallow therapy exercises – Effortful and Masako	7	7	No	Delayed swallow trigger, nil epiglottic deflection, reduced pharyngeal squeeze, penetration and aspiration during swallow. PAS 8, RS 1	No	Less oedema of supraglottic structures, nil epiglottic deflection, penetration during and post-swallow. PAS 5, RS 2
7	F	70	Systemic—pulmonary and laryngeal	0*	2 × periods of voice and swallow therapy 2016—Masako, Shaker and Effortful swallow exercises 2018—6 weeks of further swallow therapy	5		No	No	Delayed swallow trigger, significantly reduced pharyngeal squeeze, reduced hyolaryngeal excursion and reduced cricopharyngeal opening, with diffuse residue, penetration during swallow. PAS 4	No

*PAS* Penetration-Aspiration Scale, *RS* Residue Score

^*^Patient had multiple in-office vocal cord augmentation injections but no dilatation

All patients except one were seen and assessed by SLT having already had surgical intervention in the past, and prior to having a further operation. One patient was assessed just prior to his first surgical intervention. FOIS scores ranged from 5 to 7 at these initial assessments, i.e. all patients were taking a full oral diet, but some patients had to avoid certain foods or make modifications to their food or mealtimes so that they could manage more comfortably. All patients underwent an instrumental swallow assessment, either a VFS [4] or a FEES [23]. These occurred either pre- or post-operatively, or both, dependent on clinical need.

Laryngeal presentation of all patients showed supraglottic oedema and a deformed or fixed epiglottis. Only two out of seven patients had restriction of vocal fold movement, which did not correlate with increased dysphagia severity. Instrumental assessment identified a range of swallowing issues. Airway compromise and pharyngeal residue were common. Patients’ scores on the Penetration-Aspiration Scale (PAS) ranged from 1 to 8. Only one patient scored 1, which equates to ‘material does not enter the airway’. The other six patients all scored between 3 and 8, ranging from ‘material enters the airway, remains above the vocal folds and is not ejected from the airway’ to ‘material enters the airway, passes below the vocal folds, and no effort is made to eject’. The median PAS score was 5 and modal score was 8. There was no obvious trend between the type of sarcoidosis (either laryngeal or mixed) and patients’ PAS scores, nor was there any correlation between the type of surgery patients had previously undergone and their PAS scores. Penetration occurred during the swallow, with two patients also experiencing post-swallow penetration via the interarytenoid space. These patients exhibited particularly oedematous arytenoids, thereby reducing the protective filling space of the pyriform sinuses. All seven patients were either insensate to this penetration (and subsequent aspiration in three cases) or exhibited an inconsistent cough response; they were, therefore, mostly unaware of the extent of their swallowing difficulties. This is evidenced by their high scores on the FOIS, in comparison to most of their scores on the PAS which demonstrated no protective airway response to material entering the airway, i.e. silent penetration and aspiration.

Pharyngeal residue was also common, ranging from 0 to 2 on Langmore’s Residue Score, with 2 indicating moderate residue. In most cases, there was mildly reduced base of tongue to posterior pharyngeal wall squeeze, leading to some residue in the valleculae or on the pharyngeal walls. Similarly, most cases showed only partial hyolaryngeal elevation and excursion, with some residue subsequently sitting in the pyriform sinuses. Of the three patients who underwent VFS, two showed a slightly reduced cricopharyngeal opening, likely as a result of reduced hyolaryngeal movement. There was no evidence of a cricopharyngeal bar, which was the main physiological manifestation of dysphagia in the single paper in the literature that gives details on the nature of the dysphagia [8].

Two patients had instrumental assessments both pre-surgery and within a few weeks post-surgery. They both showed improvements to their PAS scores, moving from aspiration pre-operatively to just penetration post-operatively. Neither of them had undergone swallow rehabilitation at this point, so it can be hypothesised that the improvement to their swallow function was as a result of an improved breath/swallow cycle, which will be discussed further below.

SLT management was patient-specific and involved a range of input including: safe swallow advice such as modifying the consistency of food or fluids, compensatory techniques such as a chin tuck when swallowing, swallow rehabilitation exercises such as the Masako and Mendelsohn manoeuvre to address reduced base of tongue to posterior pharyngeal wall squeeze or hyolaryngeal elevation and excursion, and close surveillance, often with repeat instrumental assessments. Of the two patients who were given specific rehabilitation exercises, according to the deficits seen on their instrumental assessments, for one patient this was due to his wish to ‘future proof’ his swallow and so further instrumental assessment has not taken place at the time of writing. The other patient reported a subjective improvement in her swallow function following the exercises, although her function subsequently deteriorated again, likely as a result of fluctuating disease.

## Discussion

Laryngeal sarcoidosis is a rare condition. Dysphagia is under-reported in the literature. Whilst dysphagia is referenced as a typical symptom of laryngeal sarcoidosis, there is very limited discussion as to the nature of the dysphagia, how it presents and how it is treated. There is also no mention of SLT or swallowing rehabilitation as part of dysphagia intervention.

Our case series analysis, showing 41% of laryngeal sarcoidosis patients being actively managed at the National Centre for Airway Reconstruction were suspected of having dysphagia, is comparable to other incidence/prevalence data in the published literature which suggests dysphagic symptoms in 38% of patients with laryngeal sarcoidosis [19].

All patients in our case series were eating and drinking without the need for supplementation or tube feeding. Some were aware of their difficulties in relation to residue in the pharynx and were modifying their diet accordingly but most were unaware of the penetration and aspiration that was occurring on a consistent basis, mostly with fluids. Given their age, mobility and general good health, this did not pose an immediate risk to patients’ overall well-being. However, it is important that they understood what the potential future consequences of chronic penetration and aspiration could be, namely, repeated chest infections, gradually deteriorating lung health and the possibility of tube feeding. These sensory issues with regard to penetration and aspiration highlight the need for instrumental assessment in this population, and the danger of relying solely on bedside assessment.

A common theme for all the patients in our case series was the partial or minimal epiglottic deflection, due to physiological changes to the epiglottis as a result of the sarcoidosis. This appears to be the main reason for the penetration and aspiration observed during the swallow, and the inability to completely close off the laryngeal vestibule. The oedema of the other supraglottic structures such as the aryepiglottic folds and the arytenoids also play their part, for example, in two patients the swelling of the arytenoids meant that there was no space for even mild residue in the pyriform sinuses to collect, resulting in post-swallow penetration via the interarytenoid space.

We must also look to the nervous system to potentially explain the dysphagia observed in these patients. Nervous system involvement in systemic sarcoidosis has been described in approximately 5% of cases and 15–27% of post-mortem studies [24]. Involvement of cranial nerves IX, X and XI, which is likely to lead to dysphagia and dysphonia, is the third most common presentation after facial and optic neuropathy [25]. It is therefore possible that in some of our patients, involvement of these nerves had led to both motor and sensory deficits in the pharyngeal plexus and the larynx, contributing to reduced pharyngeal squeeze, reduced hyolaryngeal movement, and a loss of sensation in the larynx to penetration and aspiration.

The third aspect to consider with this case series of patients is their breathing pattern. The breath-swallow cycle has been well documented in terms of its function and what can happen to the swallow when this cycle is disrupted [26, 27]. For the two patients who had instrumental assessment pre- and immediately post-surgical intervention, they exhibited an improvement in their PAS scores which could be due to their improved breathing, as they still had the same physiological deficits such as an immobile epiglottis and continued oedema of the supraglottic structures.

This case series also poses the question of when SLTs should be intervening with these patients. Clearly, there is a need to see these patients pre-operatively, for the ENT surgeons to plan their intervention accordingly, and ensuring that, in dilating the airway, they are not putting the patient at greater risk of aspiration. These patients may also require monitoring post-operatively, to optimise the chance of returning to baseline swallow function. However, for those patients who are not undergoing significant surgery, the question remains as to when SLT should be seeing them for therapeutic intervention. For some of the patients in our case series, it was enough for them to know and understand the issues they were facing, and to become better-informed about possible future consequences. Others required immediate strategies and advice to help them compensate for their difficulties, as well as swallow rehabilitation exercises. One patient, the youngest in our case series at 21 years old, wished to ‘future proof’ his swallow, and to that end was given rehabilitation exercises as a preventative measure against future deterioration. With no published guidance to use in our management, the SLT team at our centre have used the principles for dysphagia management which are utilised in the head and neck cancer population [28] and extrapolated these to the laryngeal sarcoidosis population. These patients in our case series will require long-term follow-up from a dysphagia perspective, in order to build an understanding as to what further or different dysphagia management techniques may be required.

One limitation in collecting data for this case series is that these patients came to the SLT’s attention as a result of the patient mentioning some difficulty with swallowing, or because the ENT surgeons knew they were going to be undertaking surgery and wanted a baseline measure of the patient’s swallow. There could well be patients who are seen in the ENT clinic, who do not mention issues with their swallow, and who therefore never see a SLT [29]. In our case series, sensory issues are prevalent, suggesting we may be under-diagnosing dysphagia in laryngeal sarcoidosis, even at a specialist tertiary referral centre with one of the largest caseloads in the UK. Improved screening of all these patients may be required, in order to better target SLT intervention, and ensure patients’ needs are fully met. We also used a combination of instrumental swallowing assessments based on clinical need and availability in this analysis. We acknowledge that future prospective studies should consider using a single instrumental assessment modality to capture any changes in swallowing function. In addition, the amount and size of food and fluid boluses administered were patient dependent – for any future prospective research, the amount of food and fluid given would need to be standardised. Since 2019, our centre has also collected patient-reported outcome measures as routine across our caseload, including the EAT-10 [30]. This would also be useful in any future prospective study to elucidate further the patients’ perception of their swallow function, in addition to their FOIS score.

Our findings highlight a potential need for specialist dysphagia intervention and hope that this paper can open a debate on how to manage these patients. Our case series highlights the need for instrumental assessment in this population, due to the prevalence of sensory issues. This correlates with the authors' original hypothesis - that instrumental assessments would likely show a more severe dysphagia than patients were self-reporting. Further research is required to understand dysphagia management in the context of this fluctuating disease process and to understand the mechanisms underlying the swallow deficits, so that patients can achieve the best possible outcomes in the long term for their swallow function.
